# Effects of Simulated Heat Waves with Strong Sudden Cooling Weather on ApoE Knockout Mice

**DOI:** 10.3390/ijerph120605743

**Published:** 2015-05-26

**Authors:** Shuyu Zhang, Zhengzhong Kuang, Xiakun Zhang

**Affiliations:** 1 Hebei Provincial Meteorological Bureau, 178 Tiyu South Street, Shijiazhuang 050021, China; E-Mail: zhangsy@cma.gov.cn; 2 School of Applied Meteorology, Nanjing University of Information Sciences and Technology, 219 Ningliu Road, Nanjing 210044, China; E-Mail: kdzh@foxmail.com; 3 National Meteorological Centre, 46 Zhongguancun South Street, Beijing 100081, China

**Keywords:** heat wave, strong cooling, ApoE−/− mice, heat stress factor, cold stress factor, atherosclerosis, hypertensive, mechanism

## Abstract

This study analyzes the mechanism of influence of heat waves with strong sudden cooling on cardiovascular diseases (CVD) in ApoE−/− mice. The process of heat waves with strong sudden cooling was simulated with a TEM1880 meteorological-environment simulation chamber according to the data obtained at 5 a.m. of 19 June 2006 to 11 p.m. of 22 June 2006. Forty-eight ApoE−/− mice were divided into six blocks based on their weight. Two mice from each block were randomly assigned to control, heat wave, temperature drop, and rewarming temperature groups. The experimental groups were transferred into the climate simulator chamber for exposure to the simulated heat wave process with strong sudden temperature drop. After 55, 59, and 75 h of exposure, the experimental groups were successively removed from the chamber to monitor physiological indicators. Blood samples were collected by decollation, and the hearts were harvested in all groups. The levels of heat stress factors (HSP60, SOD, TNF, sICAM-1, HIF-1α), cold stress factors (NE, EPI), vasoconstrictor factors (ANGII, ET-1, NO), and four items of blood lipid (TC, TG, HDL-C, and LDL-C) were measured in each ApoE−/− mouse. Results showed that the heat waves increased the levels of heat stress factors except SOD decreased, and decreased the levels of vasoconstrictor factors and blood lipid factors except TC increased. The strong sudden temperature drop in the heat wave process increased the levels of cold stress factors, vasoconstrictor factors and four blood lipid items (except the level of HDL-C which decreased) and decreased the levels of heat stress factors (except the level of SOD which increased). The analysis showed that heat waves could enhance atherosclerosis of ApoE−/− mice. The strong sudden temperature drop during the heat wave process increased the plasma concentrations of NE and ANGII, which indicates SNS activation, and resulted in increased blood pressure. NE and ANGII are vasoconstrictors involved in systemic vasoconstriction especially in the superficial areas of the body and conducive to increased blood pressure. The increase in the blood lipid levels of TG, LDL-C, TC, and LDL-C/HDL-C further aggravated CVD. This paper explored the influence mechanism of the heat waves with sudden cooling on CVD in ApoE−/− mice.

## 1. Introduction

Cardiovascular diseases (CVDs) threaten human health with their high incidence, high disability rate, and high mortality characteristics. Coronary heart diseases, myocardial infarction, and stroke are the most serious types of CVD. The occurrence of and deaths caused by these diseases are closely related to dramatic changes in weather [1]. Atherosclerosis is the pathological basis of coronary heart diseases. Clinical studies showed that oxidative stress and inflammation responses play an important role in the occurrence and development of injury in atherosclerosis. The levels of inflammatory markers are high in patients with CVDs [2]. Animal experiments have shown that early inflammatory factors increase when heat stress is applied in rats [3]. Heat shock proteins (HSPs), also known as heat stress proteins, exhibit cell protection and other physiological functions involving high temperature, oxygen deficit, and other external environmental factors or fever; pathological stimulation of tissue trauma increased the expression of HSP. When the body encounters an adverse stimulation, HSP60, an important member of the HSP family, can be used as a specific index of the body’s ability to simulate and tolerate heat. Superoxide dismutase (SOD), an anti-oxidative biological enzyme, is important in maintaining oxidative and antioxidant balance. SOD is an accurate indicator of oxidative stimulation and vascular endothelial function and is closely related to the incidence of CVD [4]. Tumor necrosis factor (TNF) and soluble intercellular adhesion molecule 1 (sICAM-1) reflect inflammation levels in the body, and their increased expression is often associated with acute cerebrovascular disease events. In addition, hypoxia inducible factor-1α (HIF-1α) is associated with the occurrence and severity of ischemic CVD. Environmental temperature variations and oxygen partial pressure in heat waves may change the expression of HIF-1α, which is also related to the occurrence and development of coronary heart diseases. Endothelin 1 (ET-1) and nitric oxide (NO) are important factors that regulate the balance of vascular constriction and cardiovascular function in the animal body. The dynamic balance of ET-1 and NO plays an important role in regulating the function of vascular smooth muscle and vascular tension. The ratio of Et-1 and NO reflects the level of the relaxation of blood vessels and the effect of heat stimulation on animal vascular activity. Cold stimulation induces a cardiovascular system reaction by influencing the activity of the sympathetic nervous and angiotensin systems. The sympathetic nervous system response is mainly driven via the catecholamines epinephrine (EPI) and norepinephrine (NE). However repeated or long-lasting exposure to cold can affect the acclimatization processes to these reactions. EPI provides a positive inotropic effect, which enhances myocardial contractility and excitability, increases heart rate and cardiac output, and speeds up conduction. The effects of EPI on all parts of blood vessels do not only differ in terms of strength, but also in terms of constriction or relaxation. EPI can shrink the blood vessels of skin, mucous membrane, and viscera (such as the kidneys) and relax the blood vessels of coronary arteries and skeletal muscles. NE and EPI combine with α receptor to induce wide systemic vascular contraction, thereby increasing peripheral resistance. Angiotensin II (ANG II) can combine with the AT1 receptor and function in vascular smooth muscles, causing the body’s microartery systolic pressure. Under the effects of these two factors, systemic vasoconstriction leads to high blood pressure. Many studies have proven that cold stimulation can excite the sympathetic nervous and angiotensin systems, leading to elevated blood pressure [5]. Cold simulation also increases the content of NE and ANG II in blood; therefore, inhibiting cold stimulation may terminate the blood pressure increase. Some studies also found that the sympathetic nervous system elevates blood pressure by activating the renin-angiotensin system [6,7]. The commonly detected risk factors of coronary artery diseases are total cholesterol (TC), triglyceride (TG), high density lipoprotein cholesterol (HDL-C), and low density lipoprotein cholesterol (LDL-C). Currently, the relationship between blood lipid levels and CVD has been recognized by the community [8,9]. These biochemical indicators are important to determine the effects of sudden temperature drop in the heat wave process on CVD. Therefore, we selected HSP60, SOD, TNF, sICAM-1, HIF-1α, ET-1, NO, NE, EPI, ANG II, TC, TG, HDL-C, and LDL-C as detection indicators in this study.

Nanjing is the one of three big heat zones in our country that experiences frequent high temperatures and hot weather. Previous studies [10] showed that hot or cold stimulation can affect human health; nevertheless, the effects of temperature shock, which often occurs in real life, on human health are rarely studied. The statistics of the heat waves in Nanjing from 2005 to 2008, show that heat waves occurred eight times, and continuous heat waved and the heat wave processes with a strong sudden temperature drop occurred four times, respectively. We can conclude from the comparison of the two types of heat wave process that the average daily death of cardiovascular disease was 19 during the heat wave processes with strong sudden temperature drops, and during the continuous heat wave the average daily number of deaths was 17. The effects of the heat wave process with strong sudden temperature drops on cardiovascular disease therefore seems more serious than those of continuous heat waves. This fact indicated by the statistics has not been reported, which raises the question of what is the reason for this? Consequently the influence of sudden temperature drops during heat waves on human health must be evaluated. ApoE knockout mice can be used as an atherosclerosis animal model, because their pathological features are similar to those of humans [11,12], so it is suitable for the study of the mechanism of the effects the heat waves on atherosclerotic diseases. In this study, we subjected ApoE−/− mice to a typical temperature drop during the heat wave process simulated using the actual meteorological data of Nanjing. We then analyzed the effect of heat and cold stimulation on physiological indicators of ApoE−/− mice and associated the results to determine the effect on CVDs.

## 2. Experimental Section

### 2.1. Equipment and Materials

The TEM1880 meteorological-environment simulation chamber (Pulingte Co., Tianjin, China) can simulate a combined temperature-humidity-pressure test environment within the temperature range of −30 °C to 120 °C with ±0.5 °C, humidity range of 30% to 98% with ±3% RH (≥75% RH) and ±0.5% RH (<75%RH). The chamber also allows fresh air injection into the combined temperature-humidity-pressure test environment when necessary. We used a TH212 special thermal detector with a measurable temperature range of −30 °C to 50 °C with accuracies of ±0.2 °C and 0.1 °C. Medical centrifuge tubes, precision electronic balance, ultra-low temperature freezer, and ELISA were used in this experiment. The following detection kits were used: chloral hydrate, NO assay (nitrate reductase method), total SOD hydroxylamine, ET-1, s-ICAM, HSP60, TNF, EPI, NE, ANG IIs, TC, HDL-C, and LDL-C. 

### 2.2. Animals and Grouping

Forty-eight 8-week-old specific pathogen-free male ApoE−/− mice were selected for the experiments. ApoE−/− mice were produced from syngeneic C578BL/6/J mice with apolipoprotein E (ApoE) gene knockouts. As these mice present similar pathogenetic characteristics to humans, the atherosclerotic model rats are widely used for research on CVD. ApoE−/− mice were fed with high-fat diet (10% lard, 10% cholesterol, 2% cholate, and 78% basal feed) for eight weeks to develop a visible atherosclerosis model [13]. ApoE−/− mice were obtained from Vital River Laboratories (Beijing, China), and high-fat diet was purchased from Beijing Ke’ao Xieli Feed Co., Ltd. (Beijing, China).

In the breeding room, noise was maintained below 60 dB (A) and animals were kept under a circadian rhythm of 12 h/12 h (light supply from 08:00–20:00). Laboratory temperature was maintained at 27 °C, which is the average temperature of the summer heat wave for the last 10 years in Nanjing. All mice were given standard water and chow, and bedded with corn cob-like capsules that were refreshed every day. The mice were handled daily to minimize the additional effects in the experiment. The 48 ApoE−/− mice were divided into six blocks based on their weight. Each block contained eight mice. Two mice from each block were randomly assigned to the control, heat wave (group one), temperature drop (group two), and rewarming temperature groups (group three). Each group contained 12 mice.

### 2.3. Establishment of the Experimental Curve

We selected an actual heat wave phenomenon that occurred in Nanjing and simulated it for three consecutive days in the experiment. The experimental temperature simulation curve model was based on a heat wave with sudden cool weather that occurred in Nanjing from 5 a.m. on 19 June 2006 to 11 p.m. on 22 June 2006, with a continued temperature drop of more than 10 °C in 2 h.

**Figure 1:**
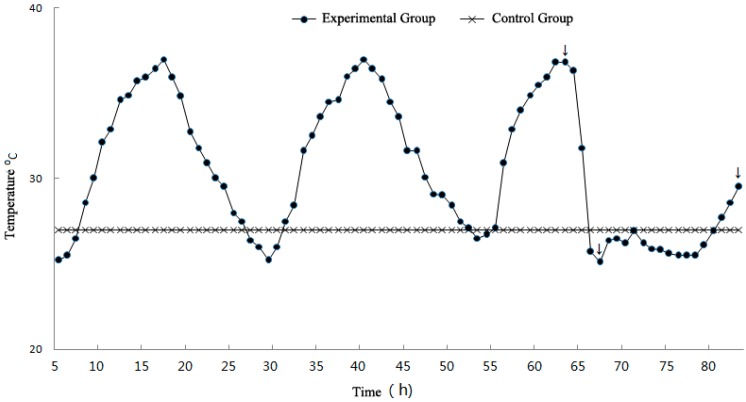
Experiment temperature curve (arrows represent sampling time points).

The simulation curve is shown in Figure 1. The experimental temperature of the control group was set at 27 °C. The experimental groups were heat wave, temperature drop, and rewarming temperature groups, which correspond to the three sample time points. The heat wave, temperature drop, and rewarming temperature groups were used to assess the effects of heat stimulation, strong sudden temperature drop, and rewarming temperature on experimental mice, respectively.

### 2.4. Experimental Process

#### 2.4.1. Preparation for the Experiment and Heat Wave Simulation 

Before the final experiment, all ApoE−/− mice were subjected to an adaptation period and fed for 8 weeks. After providing high-fat diet, all blocks of mice were divided into four groups according to the protocol described above. The heat wave models were established by manually setting the temperature evolution of the meteorological-environment simulation chamber. The experimental groups were transferred into the chamber. These groups were exposed to the heat wave process and then stimulated with high temperature, strong sudden temperature drop, and rewarming temperature. The control group was continuously fed under the adaptation period. During experiments, mice were allowed to feed and drink freely and the chamber was maintained under a circadian rhythm of 12 h/12 h (08:00–20:00). 

#### 2.4.2. Monitoring of Physiological Indicators and Sampling of Plasma and Tissue Fluid 

The entire heat wave process continued for 80 h. At the 55 h and 59 h time points as represented by the arrows in Figure 1, the heat simulation group (heat wave group) and cold simulation group (temperature drop group) were removed, respectively. Experimental group (rewarming temperature group) was removed at the 75 h time point when experimental temperature had rewarmed. The blood pressure, heart rate, body weight and rectal temperature of the experimental groups which were removed at different time points were monitored first, while the same basic physiological indicators of the control group were also monitored.

After that mice were anaesthetized through intraperitoneal injection with chloral hydrate (7% chloral hydrate and 0.3 mL/100 g). Blood samples were collected by decollation with surgical instruments and centrifuged at 3000× for 10 min. The plasma samples were stored in a refrigerator at low temperature (−20 °C) until analysis. The hearts were removed, and the weight of the cardiac apex was measured. The cardiac apex was homogenized nine times in 0.9% saline and centrifuged at 3000 rpm for 15 min. The supernatant was stored in a refrigerator at a low temperature (−20 °C) until analysis.

#### 2.4.3. Monitoring of Biochemical Indicators 

As the blood collected from mice was insufficient for all the detection experiments, we randomly selected six mice of every group for testing of HSP60, SOD, TNF, sICAM-1, HIF-1α, ET-1, NO, and NE. The six remaining mice of every group were used to detect EPI, ANGII, TC, TG, HDL-C, and LDL-C.

### 2.5. Statistical Analysis

All results were analyzed with SPSS 19.0. Data were shown as mean ± SD. All data were analyzed with paired *t*-test before and after adaptive feeding. The index results of each group were interpreted by one-way ANOVA, and the differences among treatment groups were compared with independent-sample *t*-tests. A value of *p* < 0.05 was considered statistically significant.

### 2.6. Ethic Statement

The animal protocols used in this work were evaluated and approved by the Animal Use and Ethic Committee of Hebei Provincial Meteorological Bureau (Protocol No. 2014_1). They are in accordance with *Guidance Suggestions for the Care and Use of Laboratory Animals* (issued by the Ministry of Science and Technology of the People’s Republic of China, document No. 2006_398) and the *Regulations for Laboratory Animal Management* (revised by Decree of the State Council of the People’s Republic of China, No. 638).

## 3. Results and Discussion

### 3.1. Changes in Physiological Indicators

Rectal temperature, body weight, systolic blood pressure, and heart rate are radical physiological indicators of mice and other homothermic animals as well as human in a stress affected by a temperature change. Fluctuation in rectal temperature reflects the direct effects of cold and heat stimulation on mice during the heat wave process. As shown in Table 1, changes in the body weight of ApoE−/− mice between the control and heat wave groups were not significant (*p* > 0.05) but differences between the rewarming temperature and temperature drop groups was significant (*p* < 0.05). 

The body weight of mice decreased first and then increased when the temperature was rewarmed. Mice lost 0.15 g of their body weight in the temperature drop group compared with the control group. In comparison with the rewarming temperature group, the body weight of mice increased by 1.53 and 1.95 g in the control and temperature drop groups, respectively. Rectal temperature of mice in each group evidently changed (Table 1).

**Table 1:** Comparison of rectal temperature, body weight, heart rate, and blood pressure of ApoE−/− mice (mean ± sd., *n* = 12).

Group	Control Group	Heat Wave Group	Temperature Drop Group	Rewarming Temperature Group
Rectal Temperature (°C)	37.98 ± 0.25	38.04 ± 0.25	36.98 ± 0.21 *^,#^	38.09 ± 0.32 *^,^**
Body Weight (g)	28.03 ± 2.48	28.00 ± 1.88	27.88 ± 1.33	29.83 ± 1.73 **
Heart rate (beat/min)	617 ± 40.67	575 ± 51.27	471 ± 34 *	496 ± 47 *
SBP (mmHg)	119 ± 1	118 ± 2.3	122 ± 0.67 *^,#^	117.7 ± 1.44 **

Compared with the control group, * *p* < 0.05; Compared with the heat wave group, ^#^ *p* < 0.05; Compared with the temperature drop group, ** *p* < 0.05.

In the continuous experimental process, rectal temperature increased by 0.06 °C in the heat wave group and was not significantly different from that in the control group (*p* > 0.05). As the temperature suddenly dropped, the rectal temperature of mice evidently decreased. The rectal temperature of mice reduced by 1 °C and 1.06 °C in the control and heat wave groups, respectively, and was significantly different from that in the temperature drop group (*p* < 0.05 and *p* < 0.01). This finding suggested that the heat wave process with strong sudden temperature drop could influence the rectal temperature of ApoE−/− mice. The heart rate in each group also changed as shown in Table 1. Compared with the control group, the continuous effects of heat wave on mice led to a decreased heart rate of 42 beats/min in the heat wave group. The strong sudden temperature drop led to a decreased heart rate of mice. The heart rate in the temperature drop group decreased by 146 beats/min and differed in a statistically significant way from that in the control group. The heart rate of mice gradually increased with the rewarming temperature. These results suggested that the heat wave process with strong sudden temperature drop could significantly affect the heart rate of ApoE−/− mice. The blood pressure of ApoE−/− mice in all groups also changed as shown in Table 1. In the heat wave process with strong sudden temperature drop, blood pressure increased by 3 and 4 mm Hg in the control and heat wave groups, respectively, and was statistically significantly different from that in the temperature drop group. Hence, a strong sudden temperature drop could increase blood pressure, reduce heat rate, decrease the rectal temperature of ApoE−/− mice, and only slightly affect the body weight.

### 3.2. Analysis of Heat Stimulation Factors, including HSP60, SOD, TNF, sICAM-1, and HIF-1α

As shown in Table 2, after simulation of the heat wave process, the levels of HSP60, TNF, sICAM-1, and HIF-1α (except SOD) of ApoE−/− mice showed similar changes and were significantly higher than those in the control group (Table 2). After mice were stimulated with a strong sudden temperature drop, the four indicators eased and showed varied degrees of decrease but demonstrated similar variation trends when the temperature was rewarmed.

The HSP60 level in heart tissue homogenates of mice in the heat wave group was significantly higher than that in the control group (*p* < 0.05) and increased by 1.43 ng/mL. Strong cooling led to the significant recovery of HSP60 expression levels in the heat wave group (*p* < 0.05).

Compared with the control group, the SOD expression level in the heat wave group significantly decreased (*p* < 0.01) and the value was 37.74 U/mg·prot. The strong sudden temperature drop minimally contributed to the recovery of SOD expression level in the temperature drop group. The decrease was higher (30.39 U/mg·prot) than that in the heat wave group but lower (7.35 U/mg·prot) than that in control group. The decrease was significantly different (*p* < 0.01) from that in the heat wave group. At the rewarmed temperature, the level of SOD decreased and differed significantly from the rewarming temperature group and heat wave groups. 

TNF and sICAM-1 are indicators of the degree of systemic inflammatory responses; these factors, particularly sICAM-1, increased significantly (*p* < 0.01) after experiencing heat stimulation. The level of sICAM-1 was higher in the heat wave group at 18.43 ng/L than that in the control group. TNF and sICAM-1 expression levels in the temperature group decreased significantly compared with those in the heat wave group when subjected to strong sudden temperature drop (*p* < 0.01). TNF level in the temperature drop group was significantly higher than that in the control group (*p* < 0.01). 

The expression level of HIF-1, which is closely related to the development of coronary heart diseases and other ischemic diseases, increased significantly after experiencing heat stimulation (*p* < 0.01). HIF-1α level in the heat wave group was higher at 74.34 ng/L than that in the control group, but a strong sudden temperature drop could alleviate the increase. Compared with the control group, HIF-1α levels demonstrated a significant difference at a high level (*p* < 0.01).

**Table 2:** Comparison of HSP60, SOD, TNF, sICAM-1, and HIF-α (mean ± sd., *n* = 6).

Index	Control Group	Heat Wave Group	Temperature Drop Group	Rewarming Temperature Group
HSP60 (ng/ml)	6.45 ± 0.47	7.88 ± 0.29 *	4.60 ± 0.39 *^,#^	6.26 ± 0.84 ^#^
SOD (U/mgprot)	420.19 ± 10.28	382.45 ± 7.27 *	412.84 ± 17.87 ^#^	408.43 ± 56.33 ^#^
TNF (pg/ml)	6.79 ± 0.67	7.98 ± 0.69 *	7.08 ± 0.83 *^,#^	7.26 ± 0.93 *
sICAM-1 (ng/L)	65.66 ± 2.16	84.09 ± 8.41 *	71.85 ± 3.64 *^,#^	72.89 ± 5.39 *^,#^
HIF-1α (pg/L)	745.22 ± 104.83	819.56 ± 83.59 *	652.47 ± 130.05 *^,#^	713.33 ± 97.86 ^#^

Compared with the control group, * *p* < 0.05; Compared with the heat wave group, ^#^ *p* < 0.05.

### 3.3. Analysis of Vasoconstrictor Materials

#### 3.3.1. ET-1 and NO

**Figure 2:**
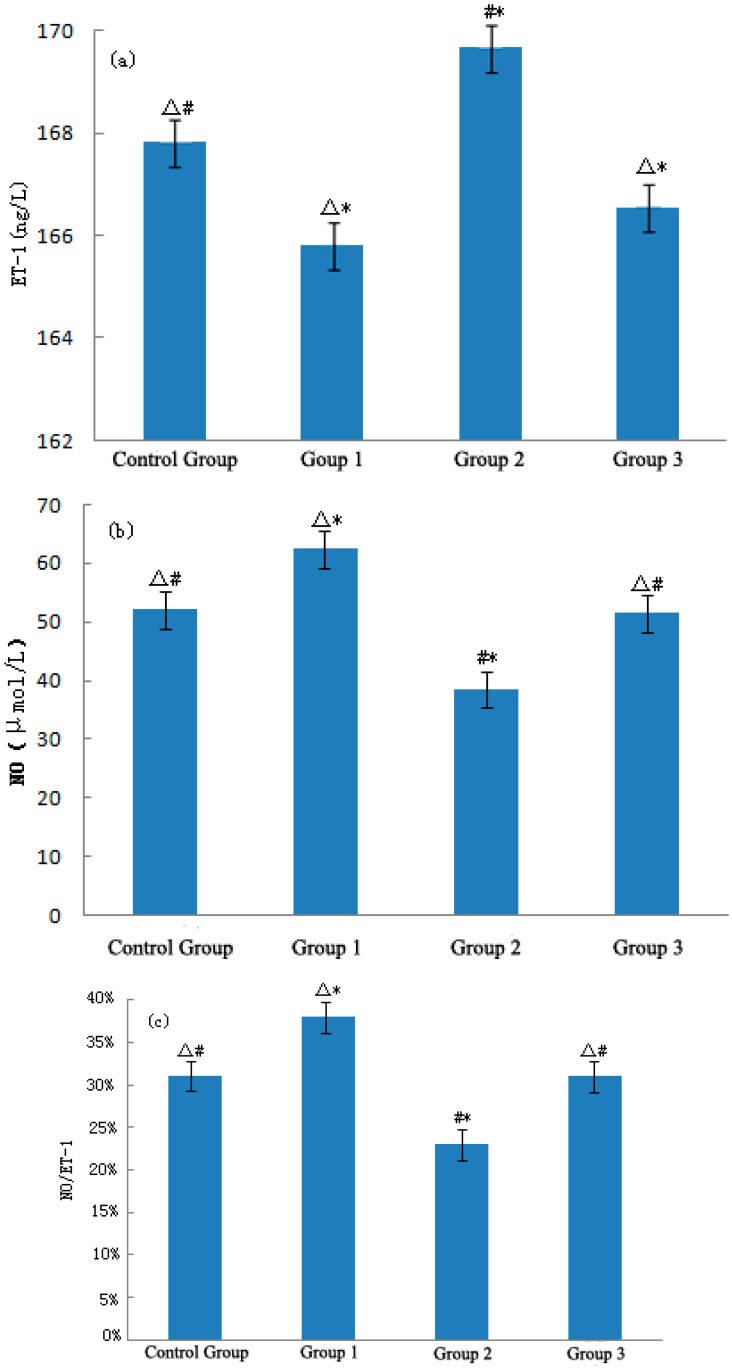
Variations of ET-1 (**a**), NO (**b**), and NO/ET-1 (**c**) in mice. Compared with the control group, * *p* < 0.01; Compared with the heat wave group, # *p* < 0.01; Compared with the temperature drop group, △ *p* < 0.01.

Figure 2a–c show the expression levels of ET-1, NO, and NO/ET-1, respectively, in mouse plasma after stimulation. During the heat wave process, the expression level of ET-1 in mouse plasma decreased significantly, with a value of 2.01 ng/L (*p* < 0.01). After experiencing the temperature drop, the level of ET-1 in the temperature drop group rapidly increased compared with the control group and heat wave groups, which significantly (*p* < 0.01) increased by 1.82 ng/L and 3.83 ng/L, respectively. As the temperature rewarmed, the expression level of ET-1 in the rewarming temperature group decreased slightly compared with that in the control and temperature drop groups. The change was statistically significant (*p* < 0.01), but was not significant between the rewarming temperature and heat wave groups (*p* > 0.05). A strong sudden temperature drop in the heat wave process affected the ET-1 levels of ApoE−/− mice. After heat stimulation, the expression level of NO in the heat wave group was significantly higher than that in the control group (*p* < 0.01) and the increase was 2.31 μmol/L. In contrast, a strong sudden temperature drop reduced the expression level of NO in the temperature drop group compared with the control and heat wave groups, which decreased by 13.69 μmol/L and 20.04 μmol/L, respectively. Both decreases were statistically significant (*p* < 0.01). 

With temperature rewarming, the level of NO in slightly increased in the rewarming temperature group but was still significantly lower than that in the control and heat wave groups (*p* < 0.01). Both heat and cold stimulation significantly influenced NO. The ratio between NO and ET-1 could reflect the level of vasodilatation and was significantly higher in the heat wave group than that in the control group (*p* < 0.01). Blood vessels tended to relax after losing heat. However, a strong temperature drop significantly decreased NO/ET-1 (*p* < 0.01) and caused rapid narrowing of blood vessels.

#### 3.3.2. NE and EPI

**Figure 3:**
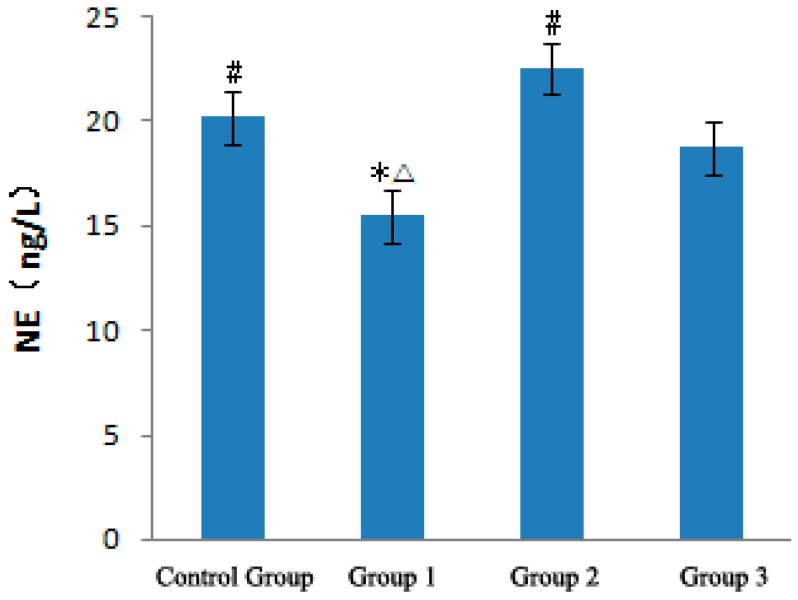
Analysis of NE in ApoE−/− mice. Compared with the control group, * *p* < 0.01; Compared with the heat wave group, # *p* < 0.01; Compared with the temperature drop group, △ *p* < 0.01.

Figure 3 presents the effects of a strong sudden temperature drop during the heat wave process on NE levels of ApoE−/− mice. In the heat wave group, the NE level was lower than that in the control group and decreased by 4.67 ng/L. With a strong sudden temperature drop, the NE level in the temperature drop group was significantly higher than that in the control and heat wave groups (*p* < 0.01), which increased by 2.38 and 7.05 ng/L, respectively. With temperature rewarming, the NE level in the rewarming temperature group began to decrease.

**Figure 4:**
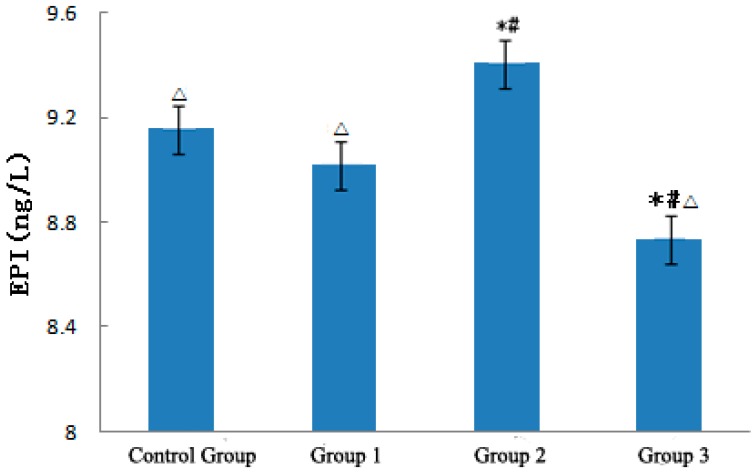
Analysis of EPI in ApoE−/− mice. Compared with the control group, * *p* < 0.01; Compared with the heat wave group, # *p* < 0.01; Compared with the temperature drop group, △ *p* < 0.01.

Figure 4 demonstrates the effect of strong sudden temperature dropping during the heat wave process on EPI level of ApoE−/− mice. EPI level in the heat wave group slightly decreased by 0.15 ng/L but was not significantly different from that in the control group.

With strong sudden temperature drop, EPI levels evidently increased in the temperature drop group and were significantly higher than those in the control and heat wave groups (p < 0.01), which increased by 0.25 and 0.39 ng/L, respectively. With temperature rewarming, the EIP level rapidly decreased in the rewarming temperature group and was significantly lower than those in the other three groups (p < 0.01). These experiment results suggest that a continuous heat wave minimally affects EPI but decreases NE levels. The strong sudden temperature drop during the heat wave process can increase NE and EPI levels, resulting in excitation of the sympathetic nervous system, inducing vasomotor effects and increased blood pressure [14,15,16,17,18], leading to CVD exacerbation [17].

#### 3.3.3. ANGII 

**Figure 5:**
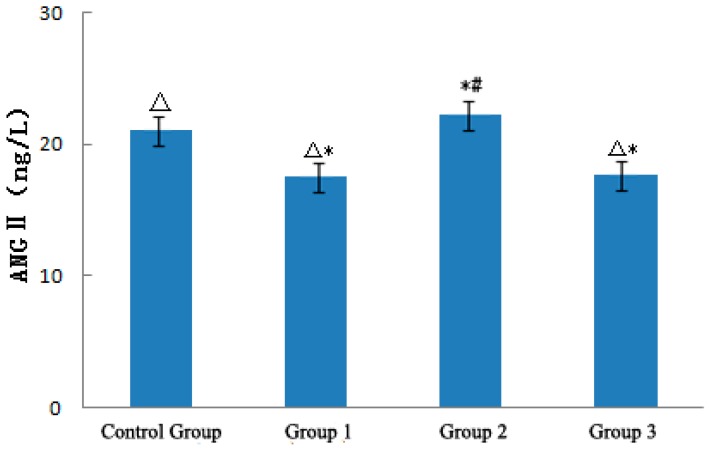
Analysis of ANG II in ApoE−/− mice.

Figure 5 shows the effect of a strong sudden temperature drop during the heat wave process on ANGII levels of ApoE−/− mice. ANGII levels in the heat wave group decreased by 3.57 ng/L compared with that in the control group. With the strong sudden temperature drop, ANGII levels in the temperature drop group were significantly higher than those in the control and heat wave groups (*p* < 0.01), which increased by 1.56 and 4.72 ng/L, respectively.

With temperature rewarming, the ANGII level in the rewarming temperature group began to decrease. ANGII levels in the rewarming temperature and temperature drop groups significantly differed from those in the control group (*p* < 0.01). However, the differences of ANGII level between the rewarming temperature and heat wave groups were not statistically significant (*p* > 0.05). These results suggested that continuous heat wave could decrease ANGII; thus, blood vessels expand [4] and become conducive to losing heat. A strong sudden temperature drop could significantly increase ANGII levels, induce vasomotor effects, and cause high blood pressure [14,15,16,17,18], leading to CVD exacerbation [17].

### 3.4. Experimental Results of Cardiovascular Risk Factors

Table 3 presents the effect of a strong sudden temperature drop during the heat wave process on blood lipid levels of ApoE−/− mice. The continuous heat wave slightly decreased the levels of HDL-C, LDL-C, TC, and LDL-C/HDL-C in the heat wave group by 0.02 mmol/L, 0.12 mmol/L, 0.05 mmol/L and 10.3%, respectively, compared with those in the control group. Only the TG level in the heat wave group was significantly higher than that in the control group (*p* < 0.01). With a strong sudden temperature drop in the heat wave process, blood lipid parameters in the temperature drop group did not present similar variation trends. HDL-C continued to decline compared with the control and heat wave groups, which decreased by 0.045 and 0.025 mmol/L, respectively. The levels of LDL-C, TC, and LDL-C/HDL-C were higher than those in the control and heat wave groups and increased by 0.14 mmol/L, 0.136 mmol/L and 32.3% compared with those in the heat group. LDL-C and LDL-C/HDL-C levels in the temperature drop group differed significantly from those in the heat wave group (*p* < 0.01). The TG level in the temperature drop group continuously increased by 1.38 and 0.26 mmol/L. The TG levels in the control group and heat wave groups significantly differed from those in the control group. With temperature rewarming, all blood lipid indicators, except HDL-C, presented a decreasing trend. HDL-C, LDL-C, and TC levels in the rewarming temperature group significantly differed from those in the temperature drop group (*p* < 0.01). However, the differences in HDL-C, LDL-C, and TC levels were not statistically significant between the rewarming temperature and heat wave groups (*p* > 0.05). Therefore, the strong sudden temperature drop during the heat wave process can increase the levels of cardiovascular blood lipid risk factors.

**Table 3:** Comparison of HDL-C, LDL-C, TC, and TG levels in ApoE−/− mice.

Index	Control Group	Heat Wave Group	Temperature Drop Group	Rewarming Temperature Group
HDL-C (mmol/L)	0.66344 ± 0.27	0.64618 ± 0.03	0.61865 ± 0.02	0.70578 ± 0.49 **
LDL-C (mmol/L)	1.66200 ± 0.09	1.54135 ± 0.16 **	1.68585 ± 0.16 ^#^	1.49493 ± 0.16 **
Tc (mmol/L)	2.25762 ± 0.05	2.20990 ± 0.22	2.34685 ± 0.11	2.04389 ± 0.93 **
T_G_ (mmol/L)	1.7155 ± 0.15 ^#,^**	2.83420 ± 0.71 *	3.09825 ± 0.93 *	2.90980 ± 0.92 *
LDL-C/HDL-C	2.505	2.402	2.725 ^#^	2.118

Compared with the control group, * *p* < 0.05; Compared with the heat wave group, # *p* < 0.05; Compared with the temperature drop group, ** *p* < 0.05.

## 4. Conclusions 

The following conclusions were established based on the experiment results:

1 A strong sudden temperature drop during a heat wave process evidently affects the physiological indicators of ApoE−/− mice. Whereas the heat wave increased the rectal temperature, the strong sudden temperature drop decreased the rectal temperature, increased blood pressure, decreased heart rate, and minimally affected the body weight.
2 With the strong sudden temperature drop during the heat wave process, the levels of HSP60, TNF, sICAM-1, and HIF-1α (except SOD) of ApoE−/− mice showed similar variation trends and were significantly higher in the heat wave group than those in the control group. After the mice experienced a strong sudden temperature drop stimulation, the four indicators were reduced to different degrees. HSP60 and HIF-α levels in the temperature drop group decreased, and the decrease was lower than those in the control and heat wave groups. TNF and sICAM-1 levels slightly decreased, and the decrease was higher than that in the control group. Therefore, the two inflammatory factors adversely affected the cardiovascular system during the strong cooling process. The expression level of SOD in ApoE−/− mice decreased in the heat wave process and was restored with a strong sudden temperature drop. The obtained value was close to the level in the control group.
3 The strong sudden temperature drop during the heat wave process could increase the level of ET-1 in ApoE−/− mice. NO was positively correlated with variations in temperature and significantly increased with the heat wave and decreased with the strong sudden temperature drop. The ratio between NO and ET-1 significantly increased as a result of the heat wave and was conducive to relaxing blood vessels and loss heat. By contrast, the strong sudden temperature drop apparently decreased NO/ET-1 levels, which was conducive to shrinking of blood vessels and maintaining the warmth of the body. The strong sudden temperature drop affected the NE levels of ApoE−/− mice. NE was inversely correlated with temperature variations, and decreased with the heat wave, and became conducive to relaxing blood vessels. Conversely, NE was increased by a strong sudden temperature drop and became conducive to shrinking blood vessels. The heat wave did not affect the level of EPI of ApoE−/− mice, but the strong sudden temperature drop increased EPI and was conducive to shrinking blood vessels. The strong sudden temperature drop during the heat wave process evidently affected the ANG II levels of ApoE−/− mice, and a continuous heat wave could decrease ANGII, causing blood vessels to expand [4], and be conducive to losing heat. Strong sudden temperature drops can significantly increase ANGII, induce vasomotor effects, and cause high blood pressure [14,15,16,17,18], leading to CVD exacerbation [17].
4 The strong sudden temperature drop during the heat wave process could affect the blood lipid levels of ApoE−/− mice. In the entire process, HDL-C of ApoE−/− mice slightly decreased and TG significantly sustained the increase. LDL-C, TC, and LDL-C/HDL-C levels were inversely correlated with temperature variations, as they decreased with the heat wave, and increased with a strong sudden temperature drop. Therefore, strong sudden temperature drops during a heat wave process can increase the levels of cardiovascular blood lipid risk factors in mice.
5 The possible mechanism of the occurrence and aggravation of CVD could be due to heat waves with strong sudden cooling weather.

A possible mechanism of the development of cardiovascular disease by strong sudden cooling weather during the heat wave process may be summarized as follows: a heat wave can increase the myocardial HSP60 content of ApoE−/− mice [19], and the excess HSP60 can activate immune cells and induce endothelial cells and macrophages to secrete numerous inflammatory cytokines, such as ICAM-1 and TNF-α [20,21]. HSP60 activates the inflammatory system *in vivo* and destroys the structure of the coronary vascular endothelial cells, thus increasing the permeability of the vascular endothelial membrane. The SOD activity of cardiac tissues decreases, which increases the fat protein oxidation in the blood. Large amounts of cholesterol are generated, accelerating cholesterol deposition in the vascular wall, which leads to atherosclerosis and the occurrence and aggravation of CVD of ApoE−/− mice [22]. With strong sudden cooling after the heat wave, the plasma concentration of NE increased, indicating that the body SNS is activated and the increased plasma concentration of ANG II also indicated that RAS was activated. The activation of these two systems will inevitably lead to an increase in blood pressure [14]. NE and ANG II are vasoconstrictors that exhibit strong vascular- contracting functions. Under the action of these two factors, systemic vasoconstriction will be more conducive to increase the blood pressure. The increase in TG, LDL-C, TC, and LDL-C/HDL-C contents could explain the elevated blood lipids, thus aggravating coronary heart diseases and may even lead to myocardial infarction.
